# Characterization of Pectin from Grape Pomace: A Comparison of Conventional and Pulsed Ultrasound-Assisted Extraction Techniques

**DOI:** 10.3390/foods11152274

**Published:** 2022-07-29

**Authors:** Mariana Spinei, Mircea Oroian

**Affiliations:** Department of Food Technologies, Food Production and Environment Safety, Faculty of Food Engineering, Stefan cel Mare University of Suceava, 720229 Suceava, Romania; m.oroian@fia.usv.ro

**Keywords:** grape pomace, pectin, characterization, rheology, comparison

## Abstract

The yield, physicochemical and rheological parameters of grape pomace pectin (Fetească Neagră and Rară Neagră) obtained by conventional extraction (CE) were compared to those acquired by pulsed ultrasound-assisted extraction (PUAE). Extraction temperature (70–90 °C), pH (1–3) and time (1–3 h) were considered as independent variables for CE, while amplitude (20–100%), pH (1–3) and time (20–60 min) for PUAE. The optimal conditions for maximum yield and physicochemical parameters of pectin samples extracted by CE were temperature of 90 °C, pH 1.9 for 164 min (9.96% yield, 79.91 g/100 g of galacturonic acid (GalA) content, 81.28% of degree of esterification (DE) and 5.52 × 10^4^ g/mol of molecular weight (M_w_) for Fetească Neagră (FN) pectin; 11.08% yield, 80.05 g/100 g of GalA content, 80.86% of DE and 5.59 × 10^4^ g/mol of M_w_ for Rară Neagră (RN) pectin), while for PUAE they were amplitude of 100%, pH 1.8 for 60 min (8.83% yield, 80.24 g/100 g of GalA content, 81.07% of DE and 4.19 × 10^4^ g/mol of M_w_ for FN pectin; 8.94% yield, 78.64 g/100 g of GalA content, 80.04% of DE and 4.23 × 10^4^ g/mol of M_w_ for RN pectin). The yield and physicochemical parameters of CE pectin were higher than PUAE pectin. The FT-IR spectra of pectin samples revealed the occurrence of polysaccharide compound, while rheology characteristics confirming its application in different food products.

## 1. Introduction

Recently, there has been a growing interest for the preservation of the environment, biodiversity and sustainability of different resources [1]. Therefore, the use of natural resources is receiving new attention as an alternative to non-renewable resources in element materials technology [2]. The efficient utilization of vegetable and fruit by-products offers immense potential for the development of different ingredients with functional properties or through the extraction of valued compounds, such as essential oils, non-starch polysaccharides, pigments and bioactive components (e.g., phenols, alkaloids, terpenes, etc.) [3]. Biopolymers (e.g., cellulose, starch, chitosan, gelatin, pectin, etc.) are the most representative organic substances that are found in natural resources [2]. Pectin is a group of polysaccharides which are consist of partly methyl-esterified galacturonic acid (GalA). The GalA units are bound to α-1,4 galacturonosyl links; this type of link is interfered with by *L*-rhamnose units which are brought by side-chains [4,5].

Generally, commercial pectin is extracted from different citrus peels, such as lemon, orange, lime, grapefruit (85.5%), apple pomace (14%) and a small proportion is attributed to sugar beet pulp (0.5%) [3]. Indeed, there are a lot of unconventional sources of pectin, including mango peels [6], banana peels [7], watermelon rinds [8], black carrot pomace [9] and grape pomace [10], mainly produced by agro-industrial processing. However, it is proper to mention that yield, physicochemical parameters, structural and functional features of pectin depend on the extraction technique applied and other characteristics (solid to liquid ratio, temperature, time, pH, solvent type) [3]. Various techniques, including conventional and non-conventional extraction methods (enzyme-, microwave-, ultrasound-assisted extraction, etc.) have been employed in order to extract pectin from different plant materials [11]. An overwhelming effort is doing based on the ethical dimension of sustainability and “green chemistry” [3]. As for the pectin extraction, conventional extraction involves high energy input, prolonged extraction time and the use of strong acid (hydrochloric, nitric and sulfuric acid), as against the principle of “green chemistry” [2].

Concerning the comparison between extraction techniques of pectin from grape pomace, no studies were found in the literature. Accordingly, regarding the lack of information, we proposed the application of grape pomace as an unconventional source of pectin. In this study, pectin was extracted from grape pomace (Fetească Neagră and Rară Neagră, *Vitis vinifera* L. varieties), by applying conventional and pulsed ultrasound extraction techniques. Therefore, the aim of this study is as follows: (1) optimizing the extraction parameters of pectin from grape pomace by using conventional and pulsed ultrasound methods; (2) understanding the combined influence of performing variables (sonication time, extraction temperature, pH and ultrasound power); and (3) comparative analysis of extraction yield, physicochemical, morphological and rheological characteristics of pectin extracted by conventional and pulsed ultrasound techniques.

## 2. Materials and Methods

### 2.1. Materials

Grape pomace was collected by processing Fetească Neagră (FN) and Rară Neagră (RN) varieties from the 2019 harvest, cultivated in the Bugeac area, Republic of Moldova. The grape pomace was dried in an oven at 50 °C until constant weight, then it was powdered and sieved in order to obtain 125–200 µm particle size using an analytical sieve shaker RetschAS 200 Basic (Retsch GmbH, Haan, Germany).

### 2.2. Methods

#### 2.2.1. Extraction of Pectin from Grape Pomace Using Conventional Extraction (CE)

About 10 g of dried grape pomace powder was weighed and placed into a Duran^®^ (DWK Life Sciences GmbH, Mainz, Germany) laboratory glass bottle and ultrapure (Milli-Q) water containing different pH values (1, 2 and 3) was added until a solid–liquid ratio of 1:10 (*w/v*) was achieved. The pH of the solvent was adjusted with citric acid. Then, the mixtures were kept in a water bath Precisdig (J.P. Selecta, Barcelona, Spain) at different temperatures (70, 80 and 90 °C) for a selected time (1, 2 and 3 h).

#### 2.2.2. Extraction of Pectin from Grape Pomace Using Pulsed Ultrasound-Assisted Extraction (PUAE)

In this stage, the effect of pulsed ultrasound-assisted treatment was studied; the dry mass (10 g) of dried grape pomace was subjected to extraction by adding 100 mL water and pH was adjusted to different values (1, 2 and 3) by citric acid. Then, the samples were heated in a water bath with constant temperature at 50 °C and were simultaneously sonicated with pulses (the pulse cycle was 1 s on and 1 s off) for different periods (20, 40 and 60 min) and amplitudes (20, 60 and 100%) (25 kHz, ultrasonic power of 200 W) using an ultrasonic bath Elma Transsonic TI-H-15 (Elma Hans Schmidbauer GmbH & Co. KG, Singen, Germany; internal dimensions: 300 × 240 × 200 mm).

#### 2.2.3. Pectin Precipitation and Purification

After extraction procedure, the mixtures were allowed to cool down to room temperature (25 °C) and separted by centrifugation (35 min at 4000 rpm), followed by the precipitation procedure with an equal volume of 96% (*v/v*) ethanol. Finally, pectin was purified with 96% (*v/v*) ethanol three times in order to achieve 1:1 ratio (*v/v*). The mixtures were kept at 4–6 °C for 12 h to accomplish the precipitation. The precipitated pectin was separated by centrifugation at 4000 rpm for 30 min. The pectin was washed 3 times with ethyl alcohol (>96%, *v/v*) and dried to a constant weight at 50 °C in an oven Zhicheng ZRD-A5055 (Zhicheng, Shanghai, China).

#### 2.2.4. Pectin Yield

Pectin yield was determined using Equation (1):

(1) $$\mathrm{Yield}\;\left(\%\right)=\frac{m_{0}}{m}\times 100$$

where: $m_{0}$—weight of dried pectin (g), m—weight of dried grape pomace powder (g) [12,13].

#### 2.2.5. Galacturonic Acid Content

The galacturonic acid (GalA) content of samples was measured using the sulfamate/*m*-hydroxydiphenyl method developed by Filisetti-Cozzi and Carpita [14] and Melton and Smith [15]. Sample preparation was made according to Miceli-Garcia [16] and Dranca and Oroian [17]. The absorbance for each sample was read at 525 nm against the reageant control with a UV-3600 Plus UV-Vis-NIR spectrophotometer (Shimadzu Corporation, Kyoto, Japan).

#### 2.2.6. Degree of Esterification

The degree of esterification (DE) of samples was determined by the titrimetric method described by Franchi [18] and Wai et al. [19]. The DE was calculated with Equation (2):

(2) $$\mathrm{DE}\;\left(\%\right)=\frac{V_{2}}{V_{1}+V_{2}}\times 100$$

where: $V_{1}$—volume of sodium hydroxide used for the first titration (mL), $V_{2}$—volume of sodium hydroxide used for the second titration (mL). The DE of pectin samples was measured in triplicate.

#### 2.2.7. Molecular Weight

Molecular weight (M_w_) of samples was carried out by high-performance size-exclusion chromatography using an HPLC system (Shimadzu Corporation, Kyoto, Japan) equipped with a LC-20 AD liquid chromatograph, SIL-20A auto sampler, a Yarra 3 µm SEC-2000 column (300 × 7.8 mm; Phenomenex, Torrance, CA, USA) and coupled with a RID-10A refractive index detector (Shimadzu Corporation, Kyoto, Japan). The samples were made according to Dranca et al. [13]. The LC solution software version 1.21 (Shimadzu Corporation, Kyoto, Japan) was utilized to collect the data.

#### 2.2.8. Color

The color of the pectin samples was analyzed in triplicate at 25 °C with a CR-400 chromameter (Konica Minolta, Tokyo, Japan). CIE L*, hue (h*_ab_) and chroma (C*_ab_) were obtained from the reflection spectra of the pectin samples with illuminant D65 and 2° observer.

#### 2.2.9. FT-IR Analysis

The samples extracted by CE and PUAE in optimal conditions (FN and RN pectin) were conducted to the FT-IR analysis using a Spectrum Two infrared spectrophotometer (PerkinElmer, Waltham, MA, USA). The distinctive spectra were registered (three scans for each sample) in the frequency range of 4000–400 cm^−1^ at a resolution of 4 cm^−1^ [20]. Omnic software (Version 9.9.473, Thermo Fisher Scientific, Waltham, MA, USA) was used to display the spectra.

#### 2.2.10. Rheological Characterization of Pectin Solutions

In order to obtain 5% (*w/w*) solutions, pectin sample extracted by CE and PUAE in optimal conditions were homogenized using Milli-Q water adjusted to pH 4 under constant stirring at 40 °C for 12 h. Then, the samples were cooled to room temperature (25 °C) and stocked under refrigeration at 4 °C for 16 h.

The dynamic viscosity of pectin solutions was determined with a Haake Mars 40 rheometer (Thermo Fisher Scientific, Waltham, MA, USA) using a cone (Ø 35 mm, 2°)—plate system. Pectin samples were let for 10 min in order to achieve the structure recovering and suitable temperature; the analysis was accomplished three times for each sample at 20 °C.

During measuring the dynamic viscosity (η, Pa·s) and shear stress (τ, Pa), the shear rate ($\dot{\gamma }$, s^−1^) was ranged between 0 and 100 s^−1^. The stress sweeps of loss modulus (G′, Pa) and elastic modulus (G″, Pa) were determined at 1 Hz for measurement of the viscoelastic region. The frequency was presented a range from 0.1 to 100 Hz and the stress was selected within the linear viscoelastic region.

The ‘creep and recovery’ analysis was measured at a constant stress of 1 Pa, which was implemented and maintained for 180 s; the stress was released to accept sample recovery for 180 s. Haake RheoWin software (Version 4.85.0000, Thermo Fisher Scientific, Waltham, MA, USA) was used to display the rheological curves.

#### 2.2.11. Statistical Analysis

In this study, a three-factor full factorial Box–Behnken design was adjusted in order to analyze and optimize the influence of the independent variables, temperature ($X_{1}$), pH ($X_{2}$) and time ($X_{3}$) on the yield, DE, GalA and M_w_ of pectin extracted under CE; amplitude ($X_{4}$), pH ($X_{5}$) and time ($X_{6}$) on the yield, DE, GalA and M_w_ of pectin extracted under PUAE. The coded levels of the variables are presented in Table 1. All graphics and calculations were accomplished utilizing the statistical software Design Expert 13 (trial version, Minneapolis, MN, USA); the analysis was rehearsed in triplicate.

The results of color, creep and recovery parameters were submitted to analysis of variance (ANOVA) using XLSTAT software (Addinsoft, New York, NY, USA). The ANOVA test was used to evaluate the difference between means at the 95% confidence level (*p* < 0.05) with Fisher’s least significant difference (LSD) procedure.

## 3. Results and Discussion

### 3.1. Model Fitting and Statistical Analysis

The conventional and pulsed ultrasound-assisted extraction (CE and PUAE, respectively) of pectin from Fetească Neagră (FN) and Rară Neagră (RN) grape pomace was modeled utilizing the Box–Behnken design with three parameters in accordance with the data presented in Appendix A. Each independent variable had three levels, as follows: temperature (70, 80 and 90 °C), time (1, 2 and 3 h) and pH (1, 2 and 3) for CE; amplitude (20, 60 and 100%), time (20, 40 and 60 min) and pH (1, 2 and 3) for PUAE. The responses of the design were extraction yield (Y, %), galacturonic acid content (GalA, g/100 g), degree of esterification (DE, %) and molecular weight (M_w_, g/mol) of pectin.

The model applied to predict the evolution of the responses was a quadratic (second order) polynomial response surface model which was used to fit the results accomplished by design; the data of the analysis of variance (ANOVA) was presented in Appendix B. In case of CE, the square polynomial equations that characterized the combined influence of temperature ($X_{1}$), pH ($X_{2}$) and time ($X_{3}$) on the yield, DE, GalA and M_w_ of extracted pectin are presented below.

(3) $$\begin{array}{cc} Y_{\text{FN-CE}}\left(\%\right)= & 8.38+1.29\times X_{1}-0.07\times X_{2}+0.56\times X_{3}-0.92\times X_{1}\times X_{2}-0.38\times X_{1}\times X_{3}-0.60\times X_{2}\times X_{3}+0.43\\ & \times X_{1}^{2}-0.96\times X_{2}^{2}-0.73\times X_{3}^{2} \end{array}$$

(4) $$\begin{array}{cc} {\mathrm{GalA}}_{\text{FN-CE}}\left(g/100\;g\right) & \\ & =74.34+2.74\cdot X_{1}-0.30\times X_{2}\times 1.15\times X_{3}-2.58\times X_{1}\times X_{2}+0.43\times X_{1}\times X_{3}-0.62\times X_{2}\times X_{3}\\ & +1.01\times X_{1}^{2}-2.43\times X_{2}^{2}-1.47\times X_{3}^{2} \end{array}$$

(5) $$\begin{array}{cc} {\mathrm{DE}}_{\text{FN-CE}}\left(\%\right) & =77.51+3.03\times X_{1}-0.50\times X_{2}+0.87\times X_{3}-2.09\times X_{1}\times X_{2}-0.63\times X_{1}\times X_{3}-0.93\times X_{2}\times X_{3}\\ & +0.89\times X_{1}^{2}-2.88\times X_{2}^{2}-1.09\times X_{3}^{2} \end{array}$$

(6) $$\begin{array}{cc} M_{w\;\text{FN-CE}}(g/\mathrm{mol}) & =53340+1625\times X_{1}+87.50\times X_{2}+762.5\times X_{3}-1000\times X_{1}\times X_{2}-350\times X_{1}\times X_{3}-975\times X_{2}\\ & \times X_{3}+442.50\times X_{1}^{2}-1332.5\times X_{2}^{2}-982.5\times X_{3}^{2} \end{array}$$

(7) $$\begin{array}{cc} Y_{\text{RN-CE}}\left(\%\right) & =9.35+1.45\times X_{1}+0.12\times X_{2}+0.48\times X_{3}-0.39\times X_{1}\times X_{2}-0.33\times X_{1}\times X_{3}-0.49\times X_{2}\times X_{3}+0.48\\ & \times X_{1}^{2}-0.90\times X_{2}^{2}-0.65\times X_{3}^{2} \end{array}$$

(8) $$\begin{array}{cc} {\mathrm{GalA}}_{\text{RN-CE}}(g/100\;g) & =72.32+4.72\times X_{1}+0.08\times X_{2}+0.51\times X_{3}-0.81\times X_{1}\times X_{2}-0.27\times X_{1}\times X_{3}-0.58\times X_{2}\\ & \times X_{3}+2.34\times X_{1}^{2}-2.03\times X_{2}^{2}-0.81\times X_{3}^{2} \end{array}$$

(9) $$\begin{array}{cc} {\mathrm{DE}}_{\text{RN-CE}}\left(\%\right) & =72.5+6.07\times X_{1}+0.85\cdot X_{2}+0.81\times X_{3}-0.53\times X_{1}\times X_{2}+0.51\times X_{1}\times X_{3}-0.87\times X_{2}\times X_{3}+1.96\\ & \times X_{1}^{2}-3.79\times X_{2}^{2}-2.86\times X_{3}^{2} \end{array}$$

(10) $$\begin{array}{cc} M_{w\;\text{RN-CE}}(g/\mathrm{mol}) & =50740+3212.5\times X_{1}+37.50\times X_{2}+775\times X_{3}-875\times X_{1}\times X_{2}-650\times X_{1}\times X_{3}-800\times X_{2}\\ & \times X_{3}+1567.5\times X_{1}^{2}-2332.5\times X_{2}^{2}-1057.5\times X_{3}^{2} \end{array}$$

For PUAE, the relationship between independent variables, amplitude ($X_{4}$), pH ($X_{5}$) and time ($X_{6}$), and the responses, yield, DE, GalA and M_w_ was performed as the following equations:

(11) $$\begin{array}{cc} Y_{\text{FN-PUAE}}\left(\%\right) & =7.16+0.78\times X_{4}-0.46\times X_{5}+1.1\times X_{6}+0.38\times X_{4}\times X_{5}+0.71\times X_{4}\times X_{6}-0.49\times X_{5}\times X_{6}\\ & +0.12\times X_{4}^{2}-0.25\times X_{5}^{2}-0.73\times X_{6}^{2} \end{array}$$

(12) $$\begin{array}{cc} {\mathrm{GalA}}_{\text{FN-PUAE}}(g/100\;g) & =69.71+4.74\times X_{4}-3.66\times X_{5}+6.05\times X_{6}+4.13\times X_{4}\times X_{5}+4.75\times X_{4}\times X_{6}-6.26\times X_{5}\\ & \times X_{6}+2.15\times X_{4}^{2}-2.86\times X_{5}^{2}-5.45\times X_{6}^{2} \end{array}$$

(13) $$\begin{array}{cc} {\mathrm{DE}}_{\text{FN-PUAE}}\left(\%\right) & =74.79+3.41\times X_{4}-2.16\times X_{5}+5.07\times X_{6}+2.04\times X_{4}\times X_{5}+3.02\times X_{4}\times X_{6}-3.19\times X_{5}\times X_{6}\\ & +0.7\times X_{4}^{2}-2.02\times X_{5}^{2}-3.66\times X_{6}^{2} \end{array}$$

(14) $$\begin{array}{cc} M_{w\;\text{FN-PUAE}}(g/\mathrm{mol}) & =37960+1850\times X_{4}-1200\times X_{5}+3300\times X_{6}+1300\times X_{4}\times X_{5}+2000\times X_{4}\times X_{6}-250\\ & \times X_{5}\times X_{6}-305\times X_{4}^{2}-805\times X_{5}^{2}-2355\times X_{6}^{2} \end{array}$$

(15) $$\begin{array}{cc} Y_{\text{RN-PUAE}}\left(\%\right) & =7.47+0.91\times X_{4}-0.44\times X_{5}+1.16\times X_{6}+0.5\times X_{4}\times X_{5}+0.6\times X_{4}\times X_{6}-0.11\times X_{5}\times X_{6}-0.48\\ & \times X_{4}^{2}+0.01\times X_{5}^{2}-0.57\times X_{6}^{2} \end{array}$$

(16) $$\begin{array}{cc} {\mathrm{GalA}}_{\text{RN-PUAE}}(g/100\;g) & =72.53+4.28\times X_{4}-1.67\times X_{5}+4.86\times X_{6}+1.22\times X_{4}\times X_{5}+1.75\times X_{4}\times X_{6}-0.18\times X_{5}\\ & \times X_{6}-1.09\times X_{4}^{2}+0.31\times X_{5}^{2}-2.81\times X_{6}^{2} \end{array}$$

(17) $$\begin{array}{cc} {\mathrm{DE}}_{\text{RN-PUAE}}\left(\%\right) & =74.52+3.74\times X_{4}-2.02\times X_{5}+3.87\times X_{6}+1.95\times X_{4}\times X_{5}+1.02\times X_{4}\times X_{6}+0.77\times X_{5}\times X_{6}\\ & -0.6\times X_{4}^{2}-1.12\times X_{5}^{2}-0.88\times X_{6}^{2} \end{array}$$

(18) $$\begin{array}{cc} M_{w\;\text{RN-PUAE}}(g/\mathrm{mol}) & \\ & =39680+2550\times X_{4}-1000\times X_{5}+5000\times X_{6}+550\times X_{4}\times X_{5}+2350\times X_{4}\times X_{6}+1050\times X_{5}\\ & \times X_{6}+1915\times X_{4}^{2}-565\times X_{5}^{2}-3965\times X_{6}^{2} \end{array}$$

### 3.2. Effect on Process Variables

#### 3.2.1. Effect of Extraction Parameters on Pectin Yield

**Figure 1:**
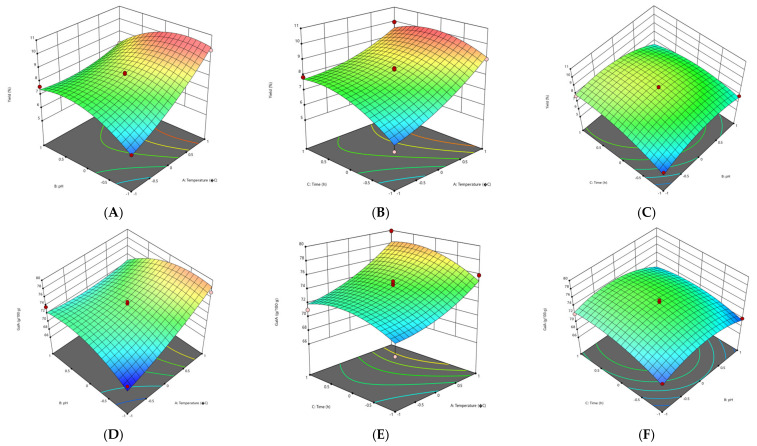

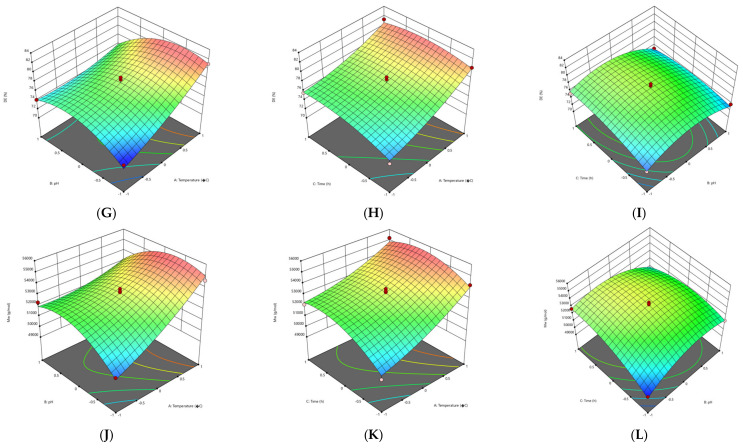
3D graphs showing the influence of extraction parameters on the yield (**A**–**C**), GalA content (**D**–**F**), DE (**G**–**I**) and M_w_ (**J**–**L**) for FN pectin extracted by CE.

**Figure 2:**
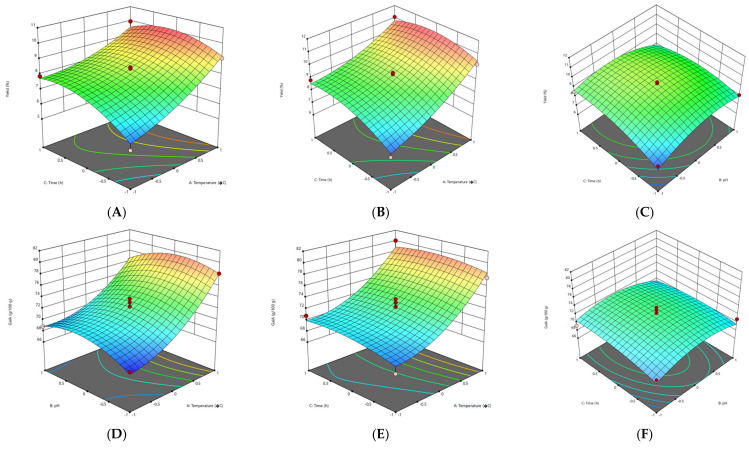

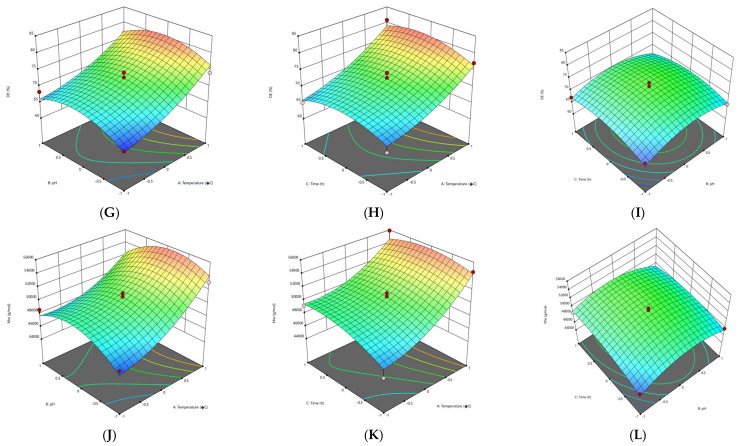
3D graphs showing the influence of extraction parameters on the yield (**A**–**C**), GalA content (**D**–**F**), DE (**G**–**I**) and M_w_ (**J**–**L**) for RN pectin extracted by CE.

**Figure 3:**
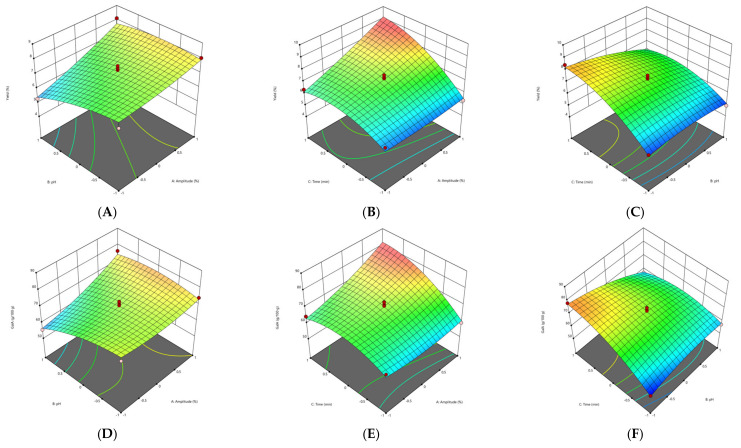

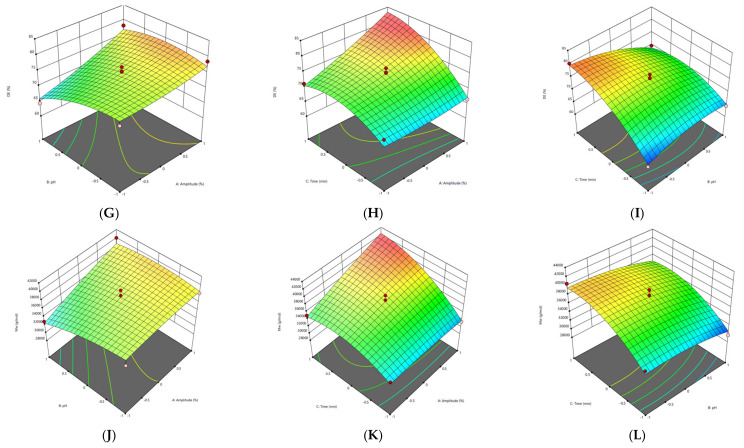
3D graphs showing the influence of extraction parameters on the yield (**A**–**C**), GalA content (**D**–**F**), DE (**G**–**I**) and M_w_ (**J**–**L**) for FN pectin extracted by PUAE.

**Figure 4:**
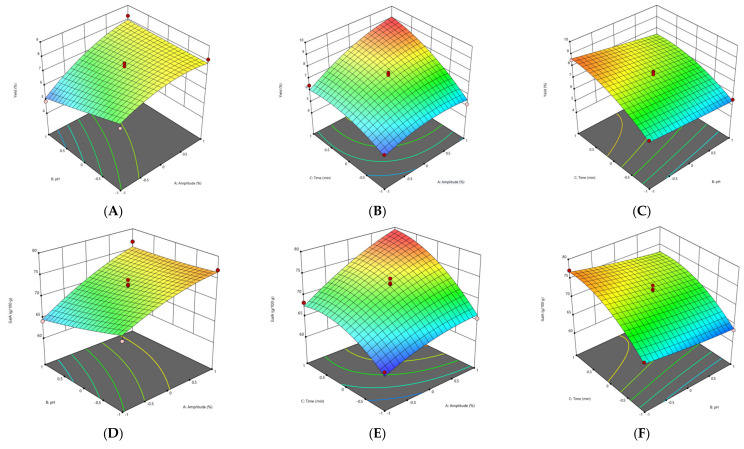

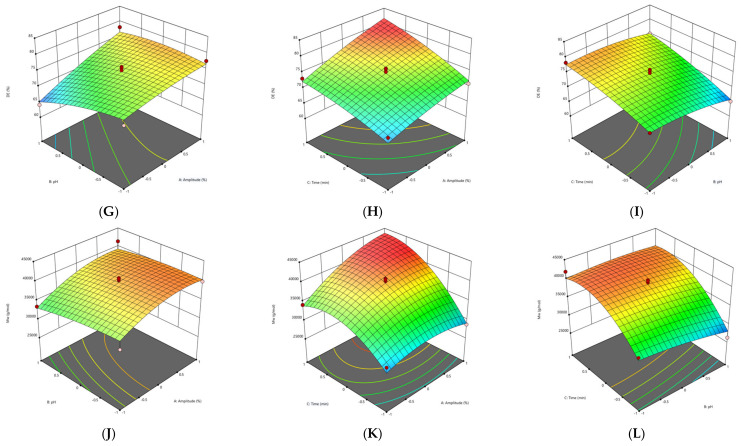
3D graphs showing the influence of extraction parameters on the yield (**A**–**C**), GalA content (**D**–**F**), DE (**G**–**I**) and M_w_ (**J**–**L**) for RN pectin extracted by PUAE.

The response surface methodology (RSM) plots (Figure 1, Figure 2, Figure 3 and Figure 4) were used for the analysis of the influence of the independent variables on the pectin characteristics (extraction yield, GalA, DE and M_w_. The three-dimensional graphics of extraction yield of Fetească Neagră (FN) and Rară Neagră (RN) pectin extracted by CE and PUAE are shown in Figure 1A–C, Figure 2A–C, Figure 3A–C and Figure 4A–C, respectively. As the results of 3D graphics and the ANOVA in Appendix B indicate, all the applied variables highly influenced the pectin yield. Extraction yield had a range between 5.43% (temperature of 70 °C, pH 2 for 1 h) and 9.96% (temperature of 90 °C, pH 2 for 3 h) for FN pectin, while for RN pectin, yield varied between 6.61% and 11.08% for similar extraction conditions by CE. Moreover, the yield of pectin obtained under PUAE presented values between 4.90% (amplitude of 60%, pH 3 for 20 min) and 8.83% (amplitude of 100%, pH 2 for 60 min) for FN pectin, while the range was between 5.16% (amplitude of 100%, pH 2 for 20 min) and 8.94% (amplitude of 100%, pH 2 for 60 min) for RN pectin.

The yield of extracted pectin (FN and RN) obtained under CE and PUAE increased when pH decreased from 3 to 1. This data is in agreement with the results obtained to extract pectin from Gac pulp [21], common fig skin [22], grapefruit peel [20] and black carrot pomace [9]. This can be related to the breakage of hydrogen bonds and ester interconnections between cell wall and pectin due to low pH, which enhances pectin extraction [21]. Moreover, these findings indicated that temperature significantly influenced the extraction yield of pectin from grape pomace. The explication is that when grape pomace pectin was extracted at high temperature, the molecular movement of the pectin was increased, resulting in morphological bundles of molecular links which would produce more junction regions to be disclosed to the solvent solution [23]. Thus, a high temperature has a greater influence on the release of soluble pectin by increasing pectin hydrolysis compared with low temperatures [23].

The other significant factor which influences the extraction yield of pectin during PUAE was the amplitude. Yousuf et al. [24] reported the highest yield of pectin from orange peels at an amplitude of 100%, pH of 1.5 and for 30 min. This can be explained by the fact that size/radius of active bubbles during cavitation are larger at a higher amplitude and thus contribute to the enhance of the reaction energy [25]. On the other hand, Wang et al. [26] reported a higher yield for grapefruit peel pectin obtained under conventional heating extraction (CHE; 23.50%) in comparison with ultrasound-assisted heating extraction (UAHE; 27.34%); the extraction time for UAHE (56 min) was 37.8% shorter than the CHE (90 min). They demonstrated that UAHE can be utilized in pectin extraction from various vegetal materials with less energy consumption and higher efficiency [26].

#### 3.2.2. Effect of Extraction Parameters on Galacturonic Acid Content

The influence of extraction characteristics on galacturonic acid (GalA) content of pectin from FN and RN pomace obtained under CE and PUAE are shown in Figure 1D–F, Figure 2D–F, Figure 3D–F and Figure 4D–F, while the results of the ANOVA are indicated in Appendix B. In agreement with the values in Appendix A, the highest GalA content was performed at the correlation of certain variables, these being an amplitude of 100%, pH 2 for 60 min (79.91 g/100 g and 80.05 g/100 g for FN and RN pectin extracted by CE, respectively), while the lowest value of GalA content was at an amplitude of 60%, pH 3 for 20 min (63.28 g/100 g for RN-PUAE) and amplitude of 60%, pH 1 for 20 min (53.17 g/100 g for FN-PUAE). There is no significant difference between GalA values of pectin (FN and RN) obtained under CE and PUAE. Figure 1D, Figure 2D, Figure 3D and Figure 4D indicate that GalA content enhanced with the amplitude, when extraction time increased. Similar results (57.25% of GalA) were obtained when a high amplitude and extraction time were applied to extract pectin from chayote (*Sechium edule*) under optimal conditions (solid-to-liquid ratio of 50 mL/g, ultrasonic time of 40 min and temperature of 70 °C) [27]. Therefore, it can be concluded that pulsed ultrasound enhances the possibility of energy stowage capacity and power for each bubble due to the lower amount of energy that is generated [25].

The interactions of amplitude–time (independent variables of PUAE) and temperature–pH (independent variables of CE) demonstrated a statistically significant influence on the GalA content of FN and RN pectin. The same data was obtained by Sabater et al. [28], who extracted pectin from artichoke by-products under ultrasound treatment and reported a GalA value of 67.85% (amplitude of 30%, pH 5 for 2 h). They noted that GalA content decreased with ultrasound-assisted extraction time and, after 6 h of treatment, it was lower than 65% [28]. In addition, the temperature also increased the GalA content through the improvement of pectin solubility with a partial enhancement of pH and rupture of the vegetal material [22].

#### 3.2.3. Effect of Extraction Parameters on Degree of Esterification

The experimental and predicted values of degree of esterification (DE) are shown in Appendix A, while the ANOVA data for DE is presented in Appendix B. The correlations among amplitude, extraction time and pH, which determine the evolution of DE of the grape pomace pectin (FN and RN) are illustrated in Figure 1G–I, Figure 2G–I, Figure 3G–I and Figure 4G–I. In agreement with the results showed in Appendix A, all pectin samples had a DE higher than 50%, ranging from 71.68% (temperature of 70 °C, pH 1 for 2 h) to 81.28% (temperature of 90 °C, pH 2 for 3 h) for FN pectin and a range of 70.12–80.86% for RN pectin extracted by CE under similar conditions; while for PUAE, values of DE were 64.37–80.24% and 63.28–78.64% for FN and RN pectin, respectively. Similar results were reported by Nguyen and Pirak [23], who obtained the highest value of DE (43.51%) for white dragon fruit peel pectin extracted by CE, while the lowest DE (34.78%) was for pectin extracted by UAE (temperature of 60 °C for 60 min). Therefore, time and temperature are important factors in the extraction of pectin; the DE of pectin obtained under CE was enhanced by increasing the time to 3 h and temperature to 90 °C. This is probably due to providing a longer extraction time, thus enhancing pectin mass transfer from particle into the solvent solution [29]. Moreover, the PUAE method had the highest influence on the DE compared to the CE technique; this can be explained by the fact that extraction conditions of PUAE (amplitude and high temperature) were severe for the extraction of grape pomace pectin which enhance the de-esterification of polygalacturonic chains [30].

On the other hand, Hosseini et al. [31] reported the highest DE value (66.67%) for sour orange peel pectin under the following conditions: ultrasound power of 100 W, time of 30 min and pH of 3.

#### 3.2.4. Effect of Extraction Parameters on Molecular Weight

The molecular weight (M_w_) of pectin is associated with its gel formation, emulsifying and stabilizing properties, which affect the utilization of pectin in the food processing [31]. For the molecular weight of pectin (FN and RN) extracted by CE and PUAE, 3D graphics are shown in Figure 1J–L, Figure 2J–L, Figure 3J–L and Figure 4J–L. The M_w_ of pectin samples ranged from 4.94 × 10^4^ g/mol (temperature of 80 °C, pH 1 for 1 h) to 5.52 × 10^4^ g/mol (temperature of 90 °C, pH 2 for 3 h) and 4.53 × 10^4^ g/mol (temperature of 70 °C, pH 2 for 1 h) to 5.59 × 10^4^ g/mol (temperature of 90 °C, pH 2 for 3 h) for FN and RN pectin extracted by CE, respectively; for PUAE, values of M_w_ were 2.96 × 10^4^—4.19 × 10^4^ g/mol and 2.63 × 10^4^—4.23 × 10^4^ g/mol for FN and RN pectin obtained under PUAE, respectively (Appendix A). The values of M_w_ of pectin depends on its natural source and extraction parameters; therefore, this range of M_w_ is lower for pectin extracted by PUAE in comparison with CE, which could be ascribed to the degradation of pectin [32]. Similar data was obtained for sugar beet pectin extracted by conventional heating compared to ultrasound-assisted extraction, 268.5 g/mol (temperature of 90 °C, pH 1 for 4 h) and 102.3 g/mol (amplitude of 96%, ultrasound frequency of 20 kHz for 10 min), respectively [32]. On the contrary, Bagherian et al. [33] reported that the M_w_ of grapefruit pectin by ultrasound-assisted extraction method (sonication time of 15 min at 70 °C) is significantly more than that obtained by conventional technique (extraction time of 90 min at 90 °C), 76.5 kDa and 18.5 kDa, respectively. It can be explained by the fact that ultrasound/pulsed ultrasound may assist with the extraction procedure through the enhancement of mass transfer and cell disruption in the outer layer of the solid material [33].

### 3.3. Optimization and Validation of Extraction Conditions

The desirability function-based method was utilized to optimize pectin yield, GalA content, DE and M_w_ concurrently. The first characteristic ($y_{i}$) was transformed in desirability function ($d_{i}$), presented in Equation (19):

(19) $$0\leq d_{i}\leq 1$$

In order to achieve the highest extraction yield, GalA content, DE and M_w_ of pectin from grape pomace (FN and RN), temperature, pH and time were optimized. The optimal conditions were the following, temperature of 90 °C, pH 1.9 for 164 min which presented a desirability function of d = 0.863 and d = 0.864 for RSM plots of FN pectin (9.96% pectin yield, 79.91 g/100 g of GalA content, 81.28% DE and 5.52 × 10^4^ g/mol of M_w_) and RN pectin (11.08% pectin yield, 80.05 g/100 g of GalA content, 80.86% DE and 5.59 × 10^4^ g/mol of M_w_) extracted under CE, respectively; amplitude of 100%, pH 1.8 for 60 min which presented a desirability function of d = 0.857 and d = 0.862 for RSM plots of FN pectin (8.83% pectin yield, 80.24 g/100 g of GalA content, 81.07% DE and 4.19 × 10^4^ g/mol of M_w_) and RN pectin (8.94% pectin yield, 78.64 g/100 g of GalA content, 80.04% DE and 4.23 × 10^4^ g/mol of M_w_) extracted under PUAE, respectively. The data was well correlated with the predicted values of responses, so the optimal conditions for CE and PUAE of pectin samples were valid.

### 3.4. Color

**Figure 5:**
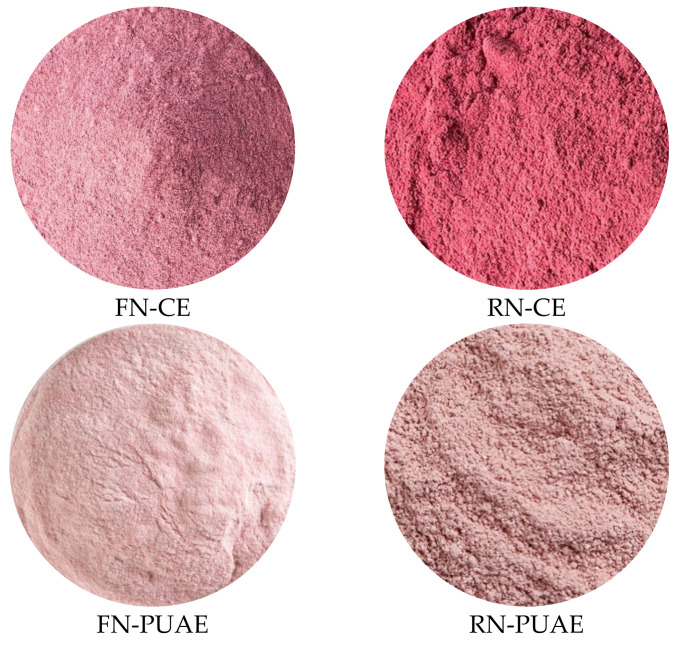
Images of pectin samples extracted from Fetească Neagră (FN) and Rară Neagră (RN) grape pomace by conventional extraction (CE) and pulsed ultrasound-assisted extraction (PUAE).

Pectin color is the first aspect and a significant factor of the appearance of emulsion or gel produced and then the one of main characteristics of the product in which it was added [32]. Different extraction parameters (pH, time, temperature and amplitude) influence the color of pectin. As can be seen in Table 2, the FN pectin extracted by PUAE had the highest lightness value (90.87), while the lowest lightness value (78.84) for FN pectin was obtained under CE. Moreover, pectin samples extracted by PUAE had the highest value of h*_ab_ (87.78 and 93.96 for RN and FN, respectively). This can be explained by the fact that high amplitude during PUAE destroyed the water-soluble compounds (e.g., pigments and polyphenols) and resulted in more purified pectin. The pectin samples obtained under CE had a lower h*_ab_ (77.28 and 74.96 for RN and FN, respectively) which could be assigned to high temperature for prolonged time applied or the pigments captured inside during precipitation [33]. Moreover, these results reflect the presence of more phenolics compounds produced by CE in comparison with PUAE (Figure 5). Since the color compounds are a result of caramelization, result of non-enzymatic Maillard reactions, and oxidation of phenolic compounds, the higher temperatures generated during cavitation-bubble collapse of the CE method can enhance the formation of highly colored pectin [34]. The same data was reported for pectin extracted from tomato waste [33], white dragon fruit peel [23] and sugar beet [35].

### 3.5. FT-IR Analysis

**Figure 6:**
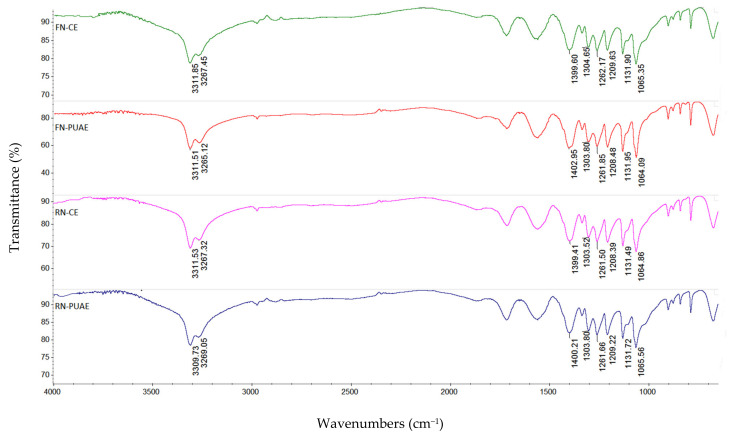
FT-IR spectra of pectin extracted from grape pomace (FN and RN) by CE and PUAE under the optimal conditions.

To investigate the different structural characteristics of pectin extracted from grape pomace (FN and RN) by utilizing conventional and pulsed ultrasound-assisted extraction (CE and PUAE, respectively), FT-IR analysis was applied. The FT-IR spectra of pectin samples obtained by CE and PUAE in the optimal conditions are presented in Figure 6. By comparing the spectra, RN pectin extracted by CE, FN pectin obtained under CE and PUAE had a peak around 3311 cm^−1^ which was attributed to –OH stretching vibration of the phenolic structures and –H bonded [36], while RN pectin extracted by PUAE had a shift at 3309 cm^−1^ was belonged to the –OH stretching absorption due to inter- and intramolecular hydrogen bonds [37]. The absorption bands at 3269 cm^−1^, 3267 cm^−1^ and 3265 cm^−1^ indicated a broad stretching of O–H [38]. The FT-IR spectra in the wavenumbers of 1410 and 800 cm^−1^ are identified to as the ‘fingerprint region’ of carbohydrates, which enables the identification of major chemical groups in different polysaccharides [10,13,39]. Therefore, the absorption peaks at 1402 cm^−1^, 1400 cm^−1^ and 1399 cm^−1^ were ascribed to the –CH bending of –CH_2_ groups, –COO symmetric stretching and asymmetric and symmetric stretching vibrations of the carboxylate anion in the side of polysaccharide, respectively [40,41]. The bands identified at 1304 cm^−1^ and 1262 cm^−1^ belonged to symmetric in–plane bending of –CH_3_, which suggested the presence of flavonoids and polyphenols [42] and C–O stretching of the ester band [43], respectively. The band at around 1210 cm^−1^ corresponded to C–O and C–C vibration bands of glycosidic bonds and pyranoid rings which are associated with the presence of GalA in pectin structure [41]. Other peaks appeared in the region of 1131 cm^−1^ and 1065 cm^−1^ which could be due to C–O–C and C–O vibration stretching in ethers or related compounds [44]. The obtained data showed that CE and PUAE had no significant influence on the main chain of pectin and could have an effect on the side chain of grape pomace pectin (FN and RN). Similar results for the pectin degradation were reported by Chen et al. [45], Ogutu and Mu [46] and Zhang et al. [47].

### 3.6. Rheological Properties

**Figure 7:**
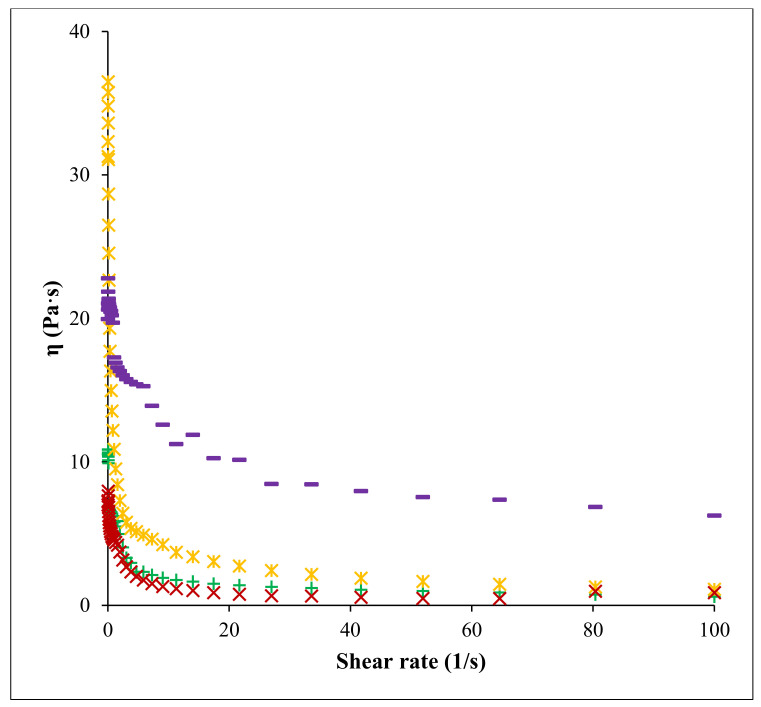
Flow curves of pectin solutions: FN pectin extracted by CE (**+**), RN pectin extracted by CE (ж), FN pectin extracted by PUAE (**×**) and RN pectin extracted by PUAE (**─**); η—dynamic viscosity, $\dot{\gamma }$ —shear rate.

The flow curves of pectin samples (FN and RN) extracted by CE and PUAE are presented in Figure 7; all curves show a non-Newtonian fluid behavior with a decrease of the dynamic viscosity and an enhancement in the shear stress. This behavior was related to a typical behavior for polysaccharide structure, where the three-dimensional network of the molecules shows a tendency to assume another conformation, align on the flow direction or dissociate, thus lowering viscosity [48]. It was noticed that RN pectin extracted by CE and PUAE had a higher dynamic viscosity than FN pectin samples, which means that source of pectin and different extraction parameters determine the pectin flow behavior [17,49]. The viscosity of RN pectin extracted by PUAE at a shear rate of 100 s^−1^ was 6.26 Pa·s which was higher than viscosity of other samples, RN-CE (1.16 Pa·s), FN-PUAE (0.88 Pa·s) and FN-CE (0.61 Pa·s). Moreover, this data was also higher than the dynamic viscosity obtained at the same shear rate (100 s^−1^) for sour orange peel pectin solution with concentration of 1.5% and 2% (less than 0.01 Pa·s) [50], 30 g/L pectin solution of finger citron pomace (0.5 Pa·s) [51] and different concentration (0.5%, 1%, 2% and 3%) of lime peel pectin solution (less than 1 Pa·s) [49]. The higher the pectin concentration is, the greater the viscosity of the pectin solution; the same tendency has been noted for citrus peel [52], cacao pod husks [53] and apple pomace [13].

**Figure 8:**
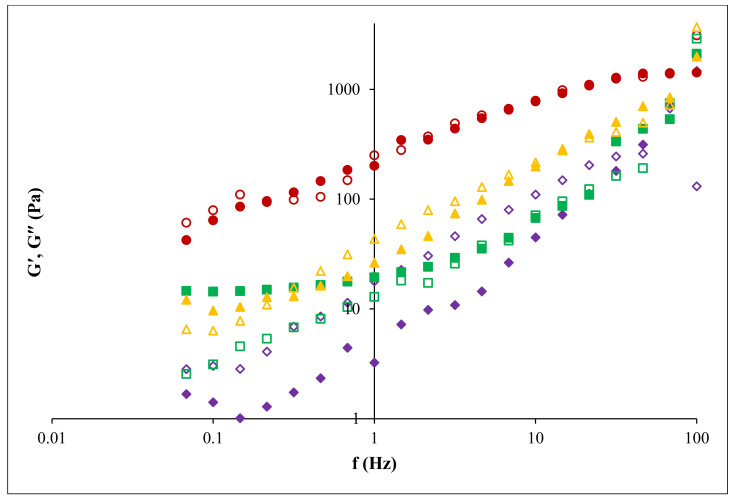
Elastic modulus (fill symbol) and loss modulus (no fill symbol) for different pectin samples: FN pectin extracted by CE (■, **□**), RN pectin extracted by CE (▲, ∆), FN pectin extracted by PUAE (**♦**, **◊**) and RN pectin extracted by PUAE (●, **○**); $G^{\prime }$—elastic modulus, $G^{\prime \prime }$ —loss modulus, f —frequency.

Dynamic viscoelastic properties of the 5% pectin solutions were analyzed by frequency sweeps acquired at 25 °C (Figure 8). Both elastic (G′) and loss (G″) moduli enhanced with the frequency, while G′ depends more on frequency than G″; all pectin samples had a higher G′ than G″ in the 0.1–100 Hz frequency domain applied until cross-over between moduli occurs. Moreover, the intersection of moduli (G′ and G″) presents a good viscoelasticity of pectin samples [54]; the same behavior was also noticed for the 5% pectin solutions from pulp of gabiroba [55], lime peel waste [49] and cacao pod husks [53]. In addition, the extraction technique had a great impact on the dynamic viscoelastic properties of pectin, and samples obtained under CE and PUAE could be considered adequate for use in the food industry.

**Figure 9:**
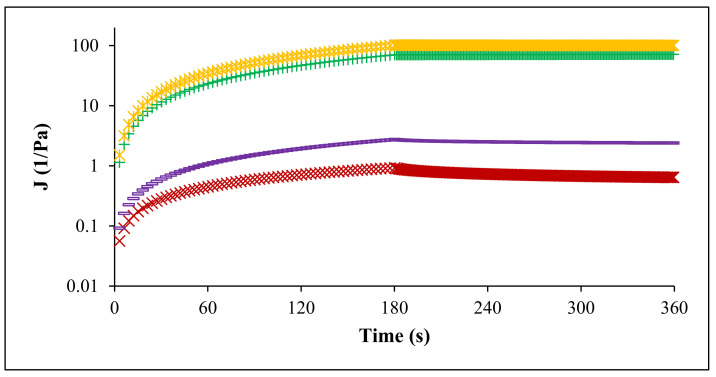
Creep and recovery test of pectin solutions: FN pectin extracted by CE (**+**), RN pectin extracted by CE (ж), FN pectin extracted by PUAE (**×**) and RN pectin extracted by PUAE (**─**); J—compliance.

In the ‘creep and recovery’ analysis (Figure 9), the pectin samples (FN and RN) were subjected to a constant stress during 360 s in order to assess the material deformation; the ‘creep’ test is from 0 to 180 s, while the ‘recovery’ test is from 180 to 360 s. All samples (FN and RN pectin extracted by CE and PUAE) manifested a non-Newtonian behavior, with a decrease of strain response during the applied stress, denoting their viscoelastic characters. The creep and recovery parameters are presented in Table 2; the equilibrium and recoverable compliance ($J_{e}$ and $J_{r}$, respectively) values for FN pectin extracted by CE and PUAE were higher than RN pectin obtained under CE and PUAE. The highest shear rate ($\dot{\gamma }$) was observed for FN pectin extracted by CE (0.115 1/s), while the lowest was obtained for RN obtained under PUAE (0.0011/s); similar data was acquired for d(log($\dot{\gamma }$)/d(log(t)).

## 4. Conclusions

Pectin was extracted from grape pomace pectin (FN and RN) by conventional extraction (CE) and pulsed ultrasound-assisted extraction (PUAE) using three independent variables, each at three levels, temperature (70, 80 and 90 °C), time (1, 2 and 3 h) and pH (1, 2 and 3); amplitude (20, 60 and 100%), time (20, 40 and 60 min) and pH (1, 2 and 3), respectively. A comprehensive comparison carried out between CE and PUAE presented that CE assured a higher pectin yield, GalA content, DE and M_w_ (9.96%, 79.91 g/100 g, 81.28% and 5.52 × 10^4^ g/mol, respectively, for FN pectin; 11.08%, 80.05 g/100 g, 80.86% and 5.59 × 10^4^ g/mol, respectively, for RN pectin) under the optimal conditions (temperature of 90 °C, pH 1.9 for 164 min for CE and amplitude of 100%, pH 1.8 for 60 min for PUAE). The pectin samples extracted by CE and PUAE under optimal conditions were analysed by FT-IR and in terms of rheological parameters. The FT-IR spectroscopy established the presence of predominantly esterified pectin in all examined samples. The viscosity of RN pectin extracted by CE and PUAE had a higher viscosity than viscosity of FN pectin. Even with this study, despite the fact that grape pomace could be an excellent pectin source, there is still a lack of studies about the application of grape pomace pectin. So, the results offer a promising field-utilized PUAE in order to reduce pectin extraction time on an industrial scale and provide its application in different food products.

## Figures and Tables

**Table 1:** Variables and levels used for Box–Behnken design.

Extraction Technique	Variables	Levels		
		−1	0	1
CE	Temperature (°C)	70	80	90
	Time (h)	1	2	3
	pH	1	2	3
PUAE	Amplitude (%)	20	60	100
	Time (min)	20	40	60
	pH	1	2	3

CE—conventional extraction, PUAE—pulsed ultrasound-assisted extraction.

**Table 2:** Color characteristics, creep and recovery parameters of pectin samples. Mean values and standard deviation, in brackets.

Sample	Color Characteristics			Creep and Recovery Parameters				
	L*	C*_ab_	h*_ab_	J_e_ (1/Pa)	J_r_ (1/Pa)	$\dot{\gamma }$ (1/s)	η (Pa·s)	$d(\log(\dot{\gamma }$))/d(log(t))·(1/s)
FN-CE	78.84 (0.16) ^c^	11.04 (0.21) ^b^	74.96 (0.28) ^d^	4.21 (0.08) ^a^	0.671 (0.31) ^b^	0.115 (0.03) ^a^	7.058 (0.28) ^b^	0.785 (0.22) ^b^
RN-CE	80.20 (0.24) ^c^	11.57 (0.18) ^a^	77.28 (0.36) ^c^	0.981 (0.26) ^b^	0.205 (0.24) ^c^	0.048 (0.08) ^c^	15.28 (0.17) ^b^	0.845 (0.08) ^a^
FN-PUAE	90.87 (0.19) ^a^	9.88 (0.29) ^c^	93.96 (0.14) ^a^	4.226 (0.18) ^a^	0.753 (0.18) ^a^	0.088 (0.12) ^b^	10.27 (0.23) ^b^	0.762 (0.18) ^c^
RN-PUAE	89.15 (0.11) ^b^	11.51 (0.27) ^a^	87.78 (0.22) ^b^	0.032 (0.05) ^c^	0.052 (0.06) ^d^	0.001 (0.02) ^d^	1450 (0.58) ^a^	0.643 (0.12) ^d^
*F*-value	198.36 ***	57.25 ***	900.26 ***	3249.34 ***	1.79 × 10^4^ ***	6836.50 ***	1268.04 ***	1.80 × 10^5^ ***

***—*p* < 0.0001, a–d—different letters in the same column indicate significant differences among samples (*p* < 0.0001) according to the LSD test with α = 0.05. FN—Fetească Neagră, RN—Rară Neagră, CE—conventional extraction, PUAE—pulsed ultrasound-assisted extraction, L*—lightness of the color, C*_ab_—chroma, h*_ab_—hue angle, J_e_—equilibrium compliance, J_r_—recoverable compliance, $\dot{\gamma }$ —shear rate, η—viscosity.

## Data Availability

The data presented in this study are available on request from the corresponding author.

## Appendix A

**Table A1:** Box–Behnken experimental design matrix with measured and predicted values.

**FN-CE**											
**Run**	**Independent Variables**			**Measured Response**				**Predicted Response**			
	**Temperature (°C)**	**pH**	**Time (h)**	**Y (%)**	**GalA (g/100 g)**	**DE (%)**	**M_w_ (g/mol)**	**Y (%)**	**GalA (g/100 g)**	**DE (%)**	**M_w_ (g/mol)**
1	80	2	2	8.26	73.18	77.49	5.29 × 10^4^	8.38	74.34	77.51	5.33 × 10^4^
2	80	3	1	6.82	70.02	72.72	5.13 × 10^4^	6.64	69.60	73.08	5.13 × 10^4^
3	80	2	2	8.47	74.23	78.16	5.32 × 10^4^	8.38	74.34	77.51	5.33 × 10^4^
4	90	2	1	9.05	75.86	80.25	5.42 × 10^4^	9.18	75.03	80.10	5.40 × 10^4^
5	80	3	3	6.21	70.19	73.06	5.07 × 10^4^	6.57	70.66	72.97	5.09 × 10^4^
6	80	2	2	8.38	74.65	77.22	5.34 × 10^4^	8.38	74.34	77.51	5.33 × 10^4^
7	80	2	2	8.42	75.07	78.65	5.35 × 10^4^	8.38	74.34	77.51	5.33 × 10^4^
8	70	2	1	5.43	68.70	72.08	4.97 × 10^4^	5.83	70.41	72.77	5.00 × 10^4^
9	70	1	2	5.75	69.13	71.68	4.99 × 10^4^	5.70	67.88	70.89	4.97 × 10^4^
10	90	3	2	8.09	71.51	75.17	5.30 × 10^4^	8.14	72.76	75.95	5.31 × 10^4^
11	70	2	3	7.87	71.03	75.63	5.21 × 10^4^	7.74	71.86	75.78	5.22 × 10^4^
12	80	2	2	8.38	74.56	76.01	5.37 × 10^4^	8.38	74.34	77.51	5.33 × 10^4^
13	80	1	1	5.94	69.42	72.14	4.94 × 10^4^	5.58	68.95	72.23	4.92 × 10^4^
14	90	2	3	9.96	79.91	81.28	5.52 × 10^4^	9.56	78.20	80.59	5.48 × 10^4^
15	80	1	3	7.75	72.10	75.20	5.27 × 10^4^	7.93	72.52	75.84	5.26 × 10^4^
16	90	1	2	9.92	77.24	81.09	5.46 × 10^4^	10.14	78.54	71.15	5.49 × 10^4^
17	70	3	2	7.63	73.75	74.13	5.23 × 10^4^	7.41	74.25	74.07	5.19 × 10^4^
**RN-CE**											
1	80	2	2	9.21	73.76	71.30	5.13 × 10^4^	9.35	72.32	72.50	5.07 × 10^4^
2	80	3	1	8.06	70.87	66.33	4.78 × 10^4^	7.92	69.62	66.76	4.78 × 10^4^
3	80	2	2	9.45	71.28	74.27	5.01 × 10^4^	9.35	72.32	72.50	5.01 × 10^4^
4	90	2	1	10.21	77.46	77.16	5.46 × 10^4^	10.48	78.33	76.34	5.46 × 10^4^
5	80	3	3	7.48	68.64	65.19	4.67 × 10^4^	7.91	69.49	66.65	4.67 × 10^4^
6	80	2	2	9.37	73.11	72.74	5.02 × 10^4^	9.35	72.32	72.50	5.07 × 10^4^
7	80	2	2	9.18	72.37	70.07	5.08 × 10^4^	9.35	72.32	72.50	5.07 × 10^4^
8	70	2	1	6.61	67.10	63.39	4.53 × 10^4^	6.91	68.33	65.24	4.66 × 10^4^
9	70	1	2	6.82	67.39	63.60	4.65 × 10^4^	6.96	67.01	63.22	4.58 × 10^4^
10	90	3	2	10.24	76.25	76.69	5.17 × 10^4^	10.10	76.63	77.07	5.23 × 10^4^
11	70	2	3	8.83	70.79	65.02	4.92 × 10^4^	8.56	69.96	65.83	4.94 × 10^4^
12	80	2	2	9.52	71.09	74.13	5.13 × 10^4^	9.35	72.32	72.50	5.07× 10^4^
13	80	1	1	7.13	69.15	64.77	4.64 × 10^4^	6.70	68.30	63.31	4.57 × 10^4^
14	90	2	3	11.08	80.05	80.86	5.59 × 10^4^	10.78	78.82	79.01	5.45 × 10^4^
15	80	1	3	8.52	69.24	67.11	4.85 × 10^4^	8.66	70.49	66.68	4.88 × 10^4^
16	90	1	2	10.49	78.12	74.15	5.31 × 10^4^	10.65	78.10	76.43	5.40 × 10^4^
17	70	3	2	8.15	68.79	68.28	4.86 × 10^4^	7.99	68.81	66.00	4.76 × 10^4^
**FN-PUAE**											
**Run**	**Independent Variables**			**Measured Response**				**Predicted Response**			
	**Amplitude (%)**	**pH**	**Time (min)**	**Y (%)**	**GalA (g/100 g)**	**DE (%)**	**M_w_ (g/mol)**	**Y (%)**	**GalA (g/100 g)**	**DE (%)**	**M_w_ (g/mol)**
1	60	2	40	7.08	72.46	75.20	3.78 × 10^4^	7.16	69.71	74.79	3.79 × 10^4^
2	100	3	40	8.11	76.33	78.12	4.01 × 10^4^	7.73	74.20	76.76	3.88 × 10^4^
3	100	2	60	8.83	80.24	81.07	4.19 × 10^4^	9.14	81.94	83.33	4.24 × 10^4^
4	20	1	40	6.71	69.91	72.91	3.62 × 10^4^	7.10	72.04	74.27	3.75 × 10^4^
5	60	2	40	7.53	70.19	76.55	3.96 × 10^4^	7.16	69.71	74.79	3.79 × 10^4^
6	60	3	60	6.24	57.09	69.75	3.59 × 10^4^	6.31	57.52	68.84	3.66 × 10^4^
7	20	2	60	6.35	64.32	71.14	3.51 × 10^4^	6.16	62.97	70.49	3.47 × 10^4^
8	60	1	20	5.12	53.17	62.11	3.32 × 10^4^	5.05	52.74	63.02	3.24 × 10^4^
9	100	1	40	8.02	74.19	78.56	3.82 × 10^4^	7.90	73.27	77.00	3.86 × 10^4^
10	60	1	60	8.42	78.15	80.23	4.05 × 10^4^	8.23	77.37	79.53	3.95 × 10^4^
11	20	2	20	5.69	62.07	68.64	3.27 × 10^4^	5.38	60.37	66.38	3.21 × 10^4^
12	60	2	40	7.25	72.62	75.03	3.73 × 10^4^	7.16	69.71	74.79	3.79 × 10^4^
13	20	3	40	5.28	55.55	64.31	3.29 × 10^4^	5.40	56.47	65.87	3.25 × 10^4^
14	60	2	40	7.36	71.10	75.19	3.84 × 10^4^	7.16	69.71	74.79	3.79 × 10^4^
15	60	2	40	6.59	62.18	71.99	3.67 × 10^4^	7.16	69.71	74.79	3.79 × 10^4^
16	100	2	20	5.33	58.99	66.50	3.15 × 10^4^	5.52	60.34	67.16	3.18 × 10^4^
17	60	3	20	4.90	57.16	64.37	2.96 × 10^4^	5.09	57.94	65.07	3.05 × 10^4^
**RN-PUAE**											
1	60	2	40	7.14	70.56	72.39	3.81 × 10^4^	7.47	72.53	74.52	3.96 × 10^4^
2	100	3	40	8.26	76.82	78.09	4.14 × 10^4^	7.97	75.58	76.46	3.93 × 10^4^
3	100	2	60	8.94	78.64	80.04	4.23 × 10^4^	9.08	79.52	81.66	4.37 × 10^4^
4	20	1	40	6.75	69.11	71.38	3.41 × 10^4^	7.04	70.35	73.01	3.62 × 10^4^
5	60	2	40	7.29	71.42	73.57	3.87 × 10^4^	7.47	72.53	74.52	3.96 × 10^4^
6	60	3	60	7.37	72.68	75.12	3.95 × 10^4^	7.51	73.04	75.13	4.02 × 10^4^
7	20	2	60	6.44	68.27	73.11	3.42 × 10^4^	6.04	67.45	72.14	3.39 × 10^4^
8	60	1	20	6.23	67.02	71.44	3.29 × 10^4^	6.09	66.66	71.43	3.22 × 10^4^
9	100	1	40	8.11	76.94	77.56	3.98 × 10^4^	7.86	76.48	76.60	4.02 × 10^4^
10	60	1	60	8.52	77.16	78.29	4.19 × 10^4^	8.63	76.74	77.63	4.01 × 10^4^
11	20	2	20	5.07	62.12	68.06	3.00 × 10^4^	4.93	61.24	66.44	2.86 × 10^4^
12	60	2	40	7.73	73.09	76.03	4.08 × 10^4^	7.47	72.53	74.52	3.96 × 10^4^
13	20	3	40	4.88	64.13	64.13	3.35 × 10^4^	5.13	64.59	65.09	3.31 × 10^4^
14	60	2	40	7.68	73.25	75.68	4.02 × 10^4^	7.47	72.53	74.52	3.96 × 10^4^
15	60	2	40	7.51	74.33	74.92	4.06 × 10^4^	1.47	72.53	74.52	3.96 × 10^4^
16	100	2	20	5.16	65.49	70.90	2.87 × 10^4^	5.56	66.31	71.87	2.90 × 10^4^
17	60	3	20	5.52	63.28	65.09	2.63 × 10^4^	5.41	63.70	65.85	2.81 × 10^4^

FN—Fetească Neagră, RN—Rară Neagră, CE—conventional extraction, PUAE—pulsed ultrasound-assisted extraction, Y—yield, GalA—galacturonic acid content, DE—degree of esterification, M_w_—molecular weight.

## Appendix B

**Table A2:** Analysis of variance (ANOVA) of constructed quadratic model.

FN-CE					RN-CE				
Source	Sum of Squares	Degree of Freedom	Mean Square	*F* -Value	Source	Sum of Squares	Degree of Freedom	Mean Square	*F* -Value
**(A) Pectin Yield (%)**									
Model	28.49	9	3.17	27.32 ***	Model	27.04	9	3.00	22.97 **
T	13.36	1	13.36	115.34 ***	T	16.85	1	16.85	128.82 ***
pH	0.05	1	0.05	0.40 ^ns^	pH	0.11	1	0.11	0.90 ^ns^
t	2.59	1	2.56	22.33 **	t	1.90	1	1.90	14.54 *
T × pH	3.44	1	3.44	29.70 ***	T × pH	0.62	1	0.62	4.77 ^ns^
T × t	0.58	1	0.58	5.05 ^ns^	T × t	0.45	1	0.45	3.48 ^ns^
pH × t	1.46	1	1.46	12.64 *	pH × t	0.97	1	0.97	7.42 *
R^2^ = 0.972					R^2^ = 0.967				
**(B) GalA (g/100 g)**									
Model	138.89	9	15.43	6.54 *	Model	226.43	9	25.16	11.96 *
T	60.01	1	60.01	25.42 **	T	178.70	1	178.70	84.93 ***
pH	0.73	1	0.73	0.31 ^ns^	pH	0.05	1	0.05	0.02 ^ns^
t	10.65	1	10.65	4.51 ^ns^	t	2.14	1	2.14	1.02 ^ns^
T × pH	26.78	1	26.78	11.34 *	T × pH	2.67	1	2.67	1.27 ^ns^
T × t	0.74	1	0.74	0.31 ^ns^	T × t	0.30	1	0.30	0.14 ^ns^
pH × t	1.58	1	1.58	0.66 ^ns^	pH × t	1.35	1	1.35	0.64 ^ns^
R^2^ = 0.893					R^2^ = 0.938				
**(C) DE (%)**									
Model	147.80	9	16.42	16.13 *	Model	422.21	9	46.91	8.95 *
T	73.63	1	73.63	72.30 ***	T	294.88	1	294.88	56.27 ***
pH	2.03	1	2.03	1.99 ^ns^	pH	5.88	1	5.88	1.12 ^ns^
t	6.09	1	6.09	5.98 ^ns^	t	5.33	1	5.33	1.02 ^ns^
T × pH	17.51	1	17.51	17.20 *	T × pH	1.14	1	1.14	0.21 ^ns^
T × t	1.59	1	1.59	1.56 ^ns^	T × t	1.07	1	1.07	0.20 ^ns^
pH × t	3.46	1	3.46	3.40 ^ns^	pH × t	3.03	1	3.03	0.57 ^ns^
R^2^ = 0.954					R^2^ = 0.920				
**(D)** **M_w_ (g/mol)**									
Model	4.67 × 10^7^	9	5.19× 10^6^	31.88 ***	Model	1.13 × 10^8^	9	8.25 × 10^7^	11.83 *
T	2.11 × 10^7^	1	2.11 × 10^7^	129.77 ***	T	8.25 × 10^7^	1	0.11 × 10^5^	66.82 ***
pH	0.61 × 10^5^	1	0.61 × 10^5^	0.37 ^ns^	pH	0.11 × 10^5^	1	4.80 × 10^6^	0.01 ^ns^
t	4.65 × 10^6^	1	4.65 × 10^6^	28.57 ***	t	4.80 × 10^6^	1	3.06 × 10^6^	3.89 ^ns^
T × pH	4.00 × 10^6^	1	4.00 × 10^6^	24.57 **	T × pH	3.06 × 10^6^	1	1.69 × 10^6^	2.48 ^ns^
T × t	4.90 × 10^6^	1	4.90 × 10^6^	3.01 ^ns^	T × t	1.69 × 10^6^	1	2.56 × 10^6^	1.37 ^ns^
pH × t	3.80 × 10^5^	1	3.80 × 10^5^	23.36 **	pH × t	2.56 × 10^6^	1	2.56 × 10^6^	2.07 ^ns^
R^2^ = 0.976					R^2^ = 0.938				
**FN-PUAE**					**RN-PUAE**				
**(A) Pectin Yield (%)**									
Model	22.55	9	2.51	14.67*	Model	24.18	9	2.69	19.54**
A	4.90	1	4.90	28.68***	A	6.72	1	6.72	48.85***
pH	1.75	1	1.75	10.24*	pH	1.60	1	1.60	11.65*
t	9.68	1	9.68	56.68***	t	10.79	1	10.79	78.47***
A × pH	0.57	1	0.57	3.38 ^ns^	A × pH	1.02	1	1.02	7.42*
A × t	2.02	1	2.02	11.81*	A × t	1.45	1	1.45	10.56*
pH × t	0.96	1	0.96	5.62 ^ns^	pH × t	0.05	1	0.05	0.35 ^ns^
R^2^ = 0.949					R^2^ = 0.961				
**(B) GalA (g/100 g)**									
Model	1074.12	9	119.35	8.63 *	Model	415.48	9	46.16	20.00 **
A	179.55	1	179.55	12.99 *	A	146.72	1	146.72	63.57 ***
pH	107.24	1	107.24	7.76 *	pH	22.18	1	22.18	9.61 *
t	292.94	1	292.94	21.19 **	t	188.57	1	188.57	81.71 ***
A × pH	68.06	1	68.06	4.92 ^ns^	A × pH	5.90	1	5.90	2.56 ^ns^
A × t	90.25	1	90.25	6.53 *	A × t	12.25	1	12.25	5.31 ^ns^
pH × t	156.88	1	156.88	11.35 *	pH × t	0.14	1	0.14	0.06 ^ns^
R^2^ = 0.917					R^2^ = 0.963				
**(C) DE (%)**									
Model	507.06	9	56.34	11.74 *	Model	297.22	9	33.02	9.47 *
A	92.82	1	92.82	19.34 **	A	111.83	1	111.83	32.06 ***
pH	37.24	1	37.24	7.76 *	pH	32.56	1	32.56	9.34 *
t	205.74	1	205.74	42.86 ***	t	119.89	1	119.89	34.37 ***
A × pH	16.65	1	16.65	3.47 ^ns^	A × pH	15.13	1	15.13	4.34 ^ns^
A × t	36.42	1	36.42	7.59 *	A × t	4.18	1	4.18	1.20 ^ns^
pH × t	40.58	1	40.58	8.45 *	pH × t	2.37	1	2.37	0.68 ^ns^
R^2^ = 0.937					R^2^ = 0.924				
**(D)** **M_w_ (g/mol)**									
Model	1.77 × 10^8^	9	1.96 × 10^7^	11.09 *	Model	3.76 × 10^8^	9	4.18 × 10^7^	11.03 *
A	2.73 × 10^7^	1	2.73 × 10^7^	15.44 *	A	5.20 × 10^7^	1	5.20 × 10^7^	13.73 *
pH	1.15 × 10^7^	1	1.15 × 10^7^	6.50 *	pH	8.00 × 10^6^	1	8.00 × 10^6^	2.11 ^ns^
t	8.71 × 10^7^	1	8.71 × 10^7^	49.13 ***	t	2.00 × 10^8^	1	2.00 × 10^8^	52.77 ***
A × pH	6.76 × 10^6^	1	6.76 × 10^6^	3.81 ^ns^	A × pH	1.21 × 10^6^	1	1.21 × 10^6^	0.32 ^ns^
A × t	1.60 × 10^7^	1	1.60 × 10^7^	9.02 *	A × t	2.21 × 10^7^	1	2.21 × 10^7^	5.83 ^ns^
pH × t	2.50 × 10^5^	1	2.50 × 10^5^	0.14 ^ns^	pH × t	4.41 × 10^6^	1	4.41 × 10^6^	1.16 ^ns^
R^2^ = 0.934					R^2^ = 0.934				

ns—*p* > 0.05, *—*p* < 0.01, **—*p* < 0.001, ***—*p* < 0.0001; FN—Fetească Neagră, RN—Rară Neagră, CE—conventional extraction, PUAE—pulsed ultrasound-assisted extraction, GalA—galacturonic acid content, DE—degree of esterification, M_w_—molecular weight, T—temperature, t—time, A—amplitude.
